# Tumor mutational profile of triple negative breast cancer patients in Thailand revealed distinctive genetic alteration in chromatin remodeling gene

**DOI:** 10.7717/peerj.6501

**Published:** 2019-02-25

**Authors:** Suvimol Niyomnaitham, Napa Parinyanitikul, Ekkapong Roothumnong, Worapoj Jinda, Norasate Samarnthai, Taywin Atikankul, Bhoom Suktitipat, Wanna Thongnoppakhun, Chanin Limwongse, Manop Pithukpakorn

**Affiliations:** 1 Department of Pharmacology, Faculty of Medicine Siriraj Hospital, Mahidol University, Bangkok, Thailand; 2 Department of Medicine, Faculty of Medicine, Chulalongkorn University, Bangkok, Thailand; 3 Department of Medicine, Faculty of Medicine Siriraj Hospital, Mahidol University, Bangkok, Thailand; 4 Siriraj Center of Research Excellence in Precision Medicine, Faculty of Medicine Siriraj Hospital, Mahidol University, Bangkok, Thailand; 5 Research Division, Faculty of Medicine Siriraj Hospital, Mahidol University, Bangkok, Thailand; 6 Department of Pathology, Faculty of Medicine Siriraj Hospital, Mahidol University, Bangkok, Thailand; 7 Department of Pathology, Queen Savang Vadhana Memorial Hospital, Thai Red Cross Society, Chonburi, Thailand; 8 Department of Biochemistry, Faculty of Medicine Siriraj Hospital, Mahidol University, Bangkok, Thailand; 9 Integrative Computational Bioscience Center, Mahidol University, Bangkok, Thailand

**Keywords:** Breast, Cancer, Thai, Asian, Genome, Histone

## Abstract

**Background:**

Triple negative breast cancer (TNBC) is a breast cancer subtype characterized by absence of both hormonal receptors and human epithelial growth factor receptor 2 (HER2). TNBC accounts for 15–20% of breast cancer. TNBC is associated with more aggressive disease and worse clinical outcome. Though the underlying mechanism of TNBC is currently unclear, the heterogeneity of clinical characteristics in various population may relate to the difference in tumor mutational profile. There were studies on TNBC gene mutations in various ethnic groups but the tumor genome data on Thai TNBC patients is currently unknown. This study aims to investigate mutational profile of Thai TNBC.

**Methods:**

The patients were Thai individuals who were diagnosed with primary breast carcinoma between 2014 and 2017. All surgically removed primary tumor tissues were carefully examined by pathologists and archived as formalin-fixed paraffin-embedded tumor. TNBC was defined by absence of hormonal receptors and HER2 by immunohistochemistry. Genomic DNA was extracted, enriched and sequenced of all exomes on the Illumina HiSeq. Genomic data were then processed through bioinformatics platform to identify genomic alterations and tumor mutational burden.

**Results:**

A total of 116 TNBC patients were recruited. Genomic analysis of TNBC samples identified 81,460 variants, of which 5,906 variants were in cancer-associated genes. The result showed that Thai TNBC has higher tumor mutation burden than previously reported data. The most frequently mutated cancer-associated gene was *TP53* similar to other TNBC cohorts. Meanwhile *KMT2C* was found to be more commonly mutated in Thai TNBC than previous studies. Mutational profile of Thai TNBC patients also revealed difference in many frequently mutated genes when compared to other Western TNBC cohorts.

**Conclusion:**

This result supported that TNBC breast cancer patients from various ethnic background showed diverse genome alteration pattern. Although *TP53* is the most commonly mutated gene across all cohorts, Thai TNBC showed different gene mutation frequencies, especially in *KMT2C*. In particular, the cancer gene mutations are more prevalent in Thai TNBC patients. This result provides important insight on diverse underlying genetic and epigenetic mechanisms of TNBC that could translate to a new treatment strategy for patients with this disease.

## Introduction

Triple negative breast cancer (TNBC) accounts for approximately 15–20% of breast cancer (Blows et al., 2010). Breast cancer patients with TNBC are not eligible for effective selective hormonal modulator or anti-HER2 treatments because of the absence of both hormonal and growth factor receptor overexpression. Chemotherapy was therefore the only available treatment of patients with TNBC. Women with TNBC displayed a clinical aggressiveness and high risk of metastasis. TNBC has also been shown to be associated with the poorer prognosis and reduced 5-year survival than other breast cancer subtypes (Malorni et al., 2012). Several studies showed the substantial racial variations of clinical behavior and prevalence of TNBC, likely owing to a heterogeneous nature of the disease. African descents are more often to present with TNBC, higher histologic grade and more aggressive breast tumors than whites (Chen & Li, 2015). Both Hispanic and African women tend to be diagnosed in more advanced stage (Banegas & Li, 2012). Studies in Asian populations demonstrated that 11% of breast cancer patients in Singapore had TNBC while this subtype accounted for 19% of Korean breast cancer patients (Rhee et al., 2008; Thike et al., 2009). The heterogeneity of TNBC on the clinical presentation as well as histologic differences may relate to variation in genetic background. The genomic profiles of TNBC in African Americans patients have been studied (Ademuyiwa et al., 2017; Huo et al., 2017). This study addressed the lack of data on the genomic profiles of TNBC in Thai and Asian population and investigated racial differences in the genetic landscape of breast cancer that could potentially identify targets suitable for specific population.

## Methods

### Study population

The study protocol was approved by the Siriraj and King Chulalongkorn Memorial Hospital Institutional Review Boards (Protocol No. 175/2559 and 642/2557). The study was conducted according to the Good Clinical Practice and the Declaration of Helsinki. All participants provided written informed consent. One hundred and sixteen Thai patients who were diagnosed with primary TNBC and treated at both hospitals between 2014 and 2017 were included. All 116 patients were female. The average age at diagnosis was 56.47 ± 11.90 years (±SD) with an age range between 25 and 79 years. The BMI was 26.28 ± 5.89 kg/m2. Majority of the patients (87%) were categorized as early stage breast cancer (20% as stage I and 67% as stage IIa). Fifty-five patients had follow-up period up to 3 years. There were six clinical relapses within the 3-years follow-up period; four cases were in stage IIa and two cases were in stage IV. One patient died during follow-up period whose cause of death did not appear to be cancer-related.

Primary tumor tissues and lymph nodes were surgically removed as a standard treatment at Siriraj and King Chulalongkorn Memorial Hospitals and examined by board-certified pathologists. The tissues were dissected for histological diagnosis and immunohistochemistry staining then archived as FFPE tumor block. TNBC subtype was defined by absence of estrogen receptor (ER), progesterone receptor (PR) and human epithelial growth factor receptor 2 (HER2) by immunohistochemistry staining with appropriate positive control. No amplification of *HER2* was also confirmed by in-situ hybridization. Independent pathologists determined only the samples of primary TNBC tumors, which had more than 50% tumor content after dissection, to be analyzed in this study. The FFPE primary tumors were sectioned into 10 micrometers using a new blade and preserved in 1.5 mL Eppendorf tubes. Blade was changed for every tissue block to prevent the contamination of DNA.

### Tumor genome sequencing and variant calls

The genomic DNA (gDNA) was extracted using Qiagen DNeasy DNA Isolation Kit (Hilden, Germany). FFPE gDNA (50–150 ng) was converted into libraries and enriched for whole exome sequencing using Agilent’s SureSelect Human All Exon V5 + UTR Sample Prep kit. Sequencing was performed on the Illumina HiSeq 2500/4000 platform with average 300× sequencing depth. Genomic data were then processed through bioinformatics platform and knowledge base (Wuxi NextCODE Genomics, Boston, MA, USA) to identify genomic alterations including single nucleotide polymorphisms/substitutions (SNPs) and small insertions/deletions (indels). A threshold of 5% allelic fraction was used for SNPs and indels. Any variants presented at allele frequency above 1% in dbSNP, 1,000 Genomes and ExAC databases were removed. To assess somatic status of mutations in a tumor-only setting, we used both MuTect2 (Broad Institute, Boston, MA, USA) and VarScan2 (Washington University, St. Louis, MO, USA) on the aligned sequence data to determine somatic variants. All variants are further annotated with the extensive pipeline including COSMIC data annotations. Additional annotation includes HGMD Professional, ClinVar, OMIM and multiple missense functional predictors including polyphen2, SIFT, LRT, MutationTaster, MutationAssessor and CADD. The effect of the sequence variants on all protein-coding genes in the RefSeq database was further annotated using the Variant Effect Predictor, which predicts the consequence of each sequence variant on all neighboring RefSeq genes based on a set of 35 consequence terms defined by the Sequence Ontology (McLaren et al., 2016). Only variants predicted to cause strong and moderate alteration on gene functions, such as stop gained/lost variants, frameshift, indels, donor/acceptor splice variants, initiator codon variants, missense variants, in-frame indels and splice region variants were selected for analysis. Finally, the results were manually reviewed by molecular geneticists. These analysis methods applied to the cancer genome atlas (TCGA) tumor variants and expression data have distinguished different molecular or histologic subtypes of breast cancer at over 97% accuracy.

### Data comparison with other breast cancer cohorts

To compare frequency of cancer gene alterations between this study and previously published data, breast cancer mutation data from TCGA, METABRIC and French cohorts were downloaded from the cBioPortal for Cancer Genomics (http://www.cbioportal.org). TCGA cohort consisted of cancer genome data form primary breast cancer patients in the United States (Cancer Genome Atlas Network, 2012). METABRIC cohort data were collected from primary breast cancer patients in the United Kingdom and Canada (Pereira et al., 2016). French cohort data were primarily from the patients with metastatic breast cancers from four different prospective trials in France (Lefebvre et al., 2016). Cancer gene mutation data from TCGA database were selected only from breast invasive ductal carcinoma that was classified as PAM50 basal subtype, which is closely related to triple negative subtype of breast cancer, while data from METABRIC and French cohorts were selected from samples with negative ER, PR and HER2, similar to this study. The comparison of mutation frequency of each cancer genes was trimmed to 173 genes due to data limitation from 173-gene panel in METABRIC cohort.

### Statistical analysis and data visualization

Genomic characteristics were compared across cohorts using one-way analysis of variance for continuous variables. The prevalence of somatic mutations was compared across cohorts. Descriptive statistics was used to show the average age and BMI of population. Statistical analyses were conducted using SAS software (version 9.4; SAS Institute, Cary, NC, USA). A two-tailed *p* value less than 0.05 was considered significant. Landscape of co-occurrence and mutual exclusion of cancer gene mutations was generated with OncoPrinter (version 1.0.1; cBioPortal for Cancer Genomics, New York, NY, USA). Mutational spectrum of three most commonly mutated genes in lollipop plot format was generated with MutationMapper (version 1.0.1; cBioPortal for Cancer Genomics, New York, NY, USA) (Gao et al., 2013).

## Results

### Comparison of gene alterations between Thai TNBC and other TNBC cohorts

**Figure 1:**
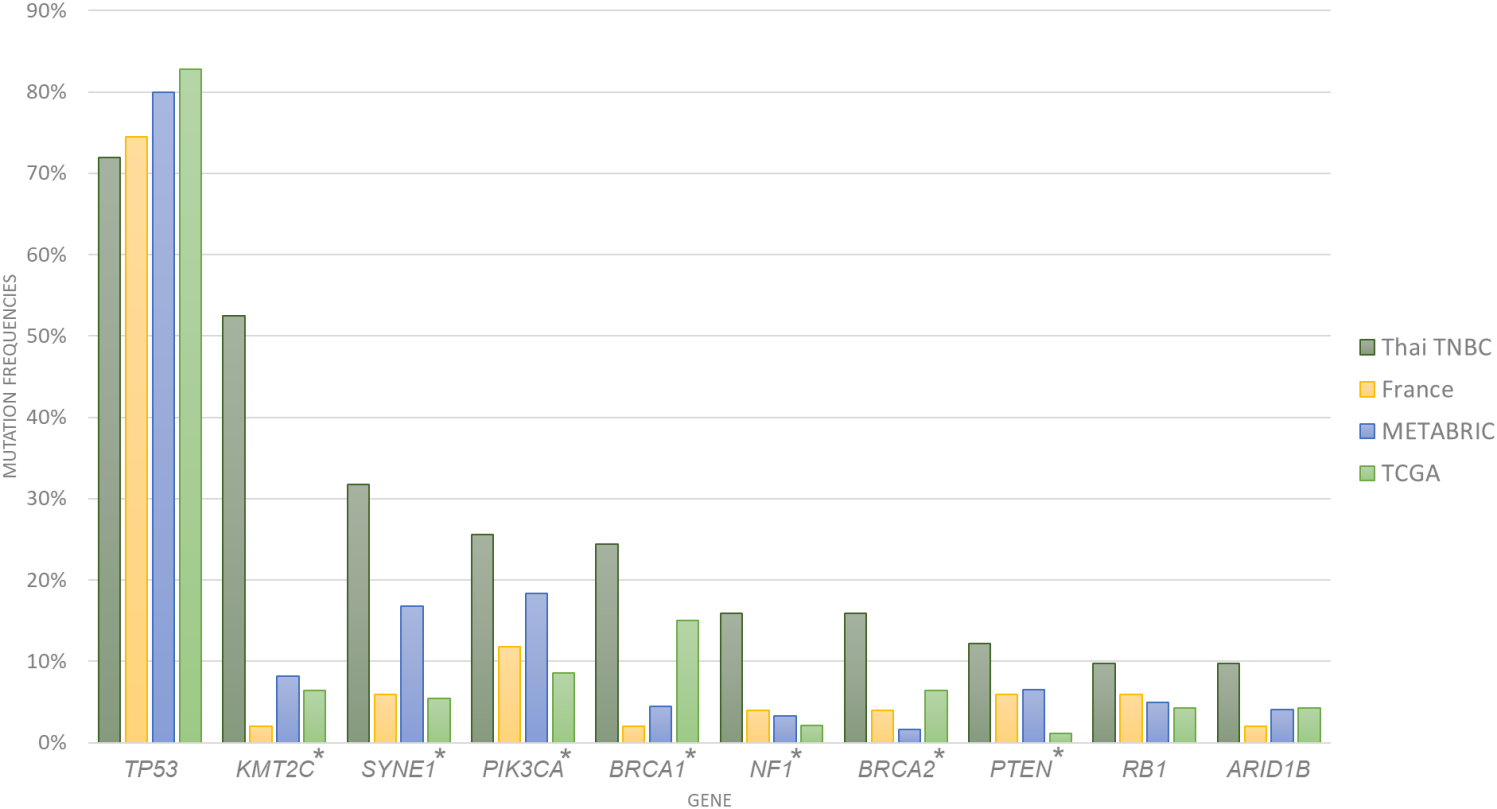
Somatic mutation frequencies among four TNBC cohorts. Bar chart showed 10 most commonly mutated in Thai TNBC compared to TCGA, METABRIC and French cohorts. * indicated the difference was statistically significant.

A total of 1,088,237 variants were detected from the whole exome sequencing data of 116 patients. Variant filtering with MuTect2 and VarScan2 identified 81,460 somatic variants that passed data analysis algorithms. When all 969 known or potential cancer-associated genes were examined, only 5,906 somatic variants were identified. We found an average of 222 variants per sample (range 104–388 variants) with an average of 20 altered cancer genes (range 5–35 genes). The 10 most commonly mutated genes were *TP53, KMT2C, SYNE1, PIK3CA, BRCA1, NF1, BRCA2, PTEN, RB1* and *ARID1B* (Fig. 1; Tables 1 and 2). Compared to the cancer gene mutation frequencies from TCGA, METABRIC and French cohorts, Thai TNBC had significantly higher mutation frequencies in *KMT2C, SYNE1, PIK3CA, NF1, PTEN, BRCA1* and *BRCA2*. On the contrary, significant differences were observed only in *SYNE1* and *BRCA1* among TCGA, METABRIC and French cohorts.

**Table 1:** Frequencies of ten most commonly mutated genes in Thai TNBC compared to other cohorts.

Gene	Thai TNBC (*n* = 116) (%)	TCGA (*n* = 93) (%)	METABRIC (*n* = 245) (%)	French (*n* = 51) (%)	Pearson chi square (*p* value)
*TP53*	75.86	82.80	80.00	74.51	0.519
*KMT2C*	57.76	6.45	8.16	1.96	<0.001
*SYNE1*	31.71	5.38	16.73	5.88	<0.001
*PIK3CA*	23.28	8.60	18.37	11.76	0.027
*BRCA1*	21.55	15.05	4.49	1.96	<0.001
*BRCA2*	18.10	6.45	1.63	3.92	<0.001
*NF1*	14.66	2.15	3.27	3.92	<0.001
*PTEN*	11.21	1.08	6.53	5.88	0.033
*RB1*	10.34	4.30	4.90	5.88	0.19
*ARID1B*	6.90	4.30	4.08	1.96	0.495

**Note:**

*n* indicated number of patients in each cohort.

**Table 2:** Frequencies of mutated genes among three published cohorts.

Gene	TCGA (*n* = 93) (%)	METABRIC (*n* = 245) (%)	French (*n* = 51) (%)	Pearson chi square (*p* value)
*TP53*	82.80	80.00	74.51	0.494
*KMT2C*	6.45	8.16	1.96	0.278
*SYNE1*	5.38	16.73	5.88	0.006
*PIK3CA*	8.60	18.37	11.76	0.063
*BRCA1*	15.05	4.49	1.96	0.001
*BRCA2*	6.45	1.63	3.92	0.068
*NF1*	2.15	3.27	3.92	0.812
*PTEN*	1.08	6.53	5.88	0.123
*RB1*	4.30	4.90	5.88	0.915
*ARID1B*	4.30	4.08	1.96	0.749

**Note:**

*n* indicated number of patients in each cohort.

### Driver gene analysis

**Figure 2:**
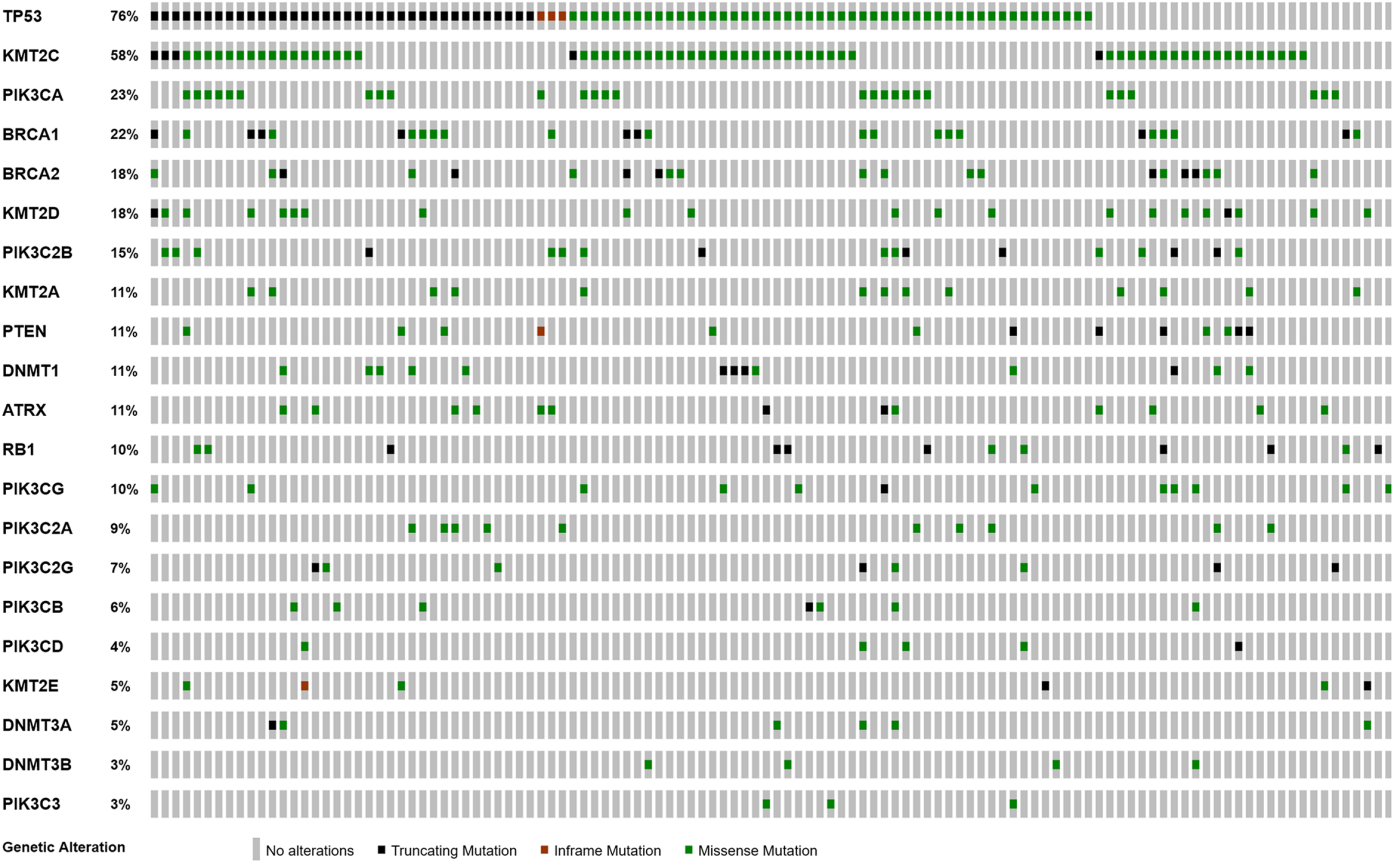
Landscape of co-occurrence and mutual exclusion of cancer gene mutations in Thai TNBC.

**Figure 3:**
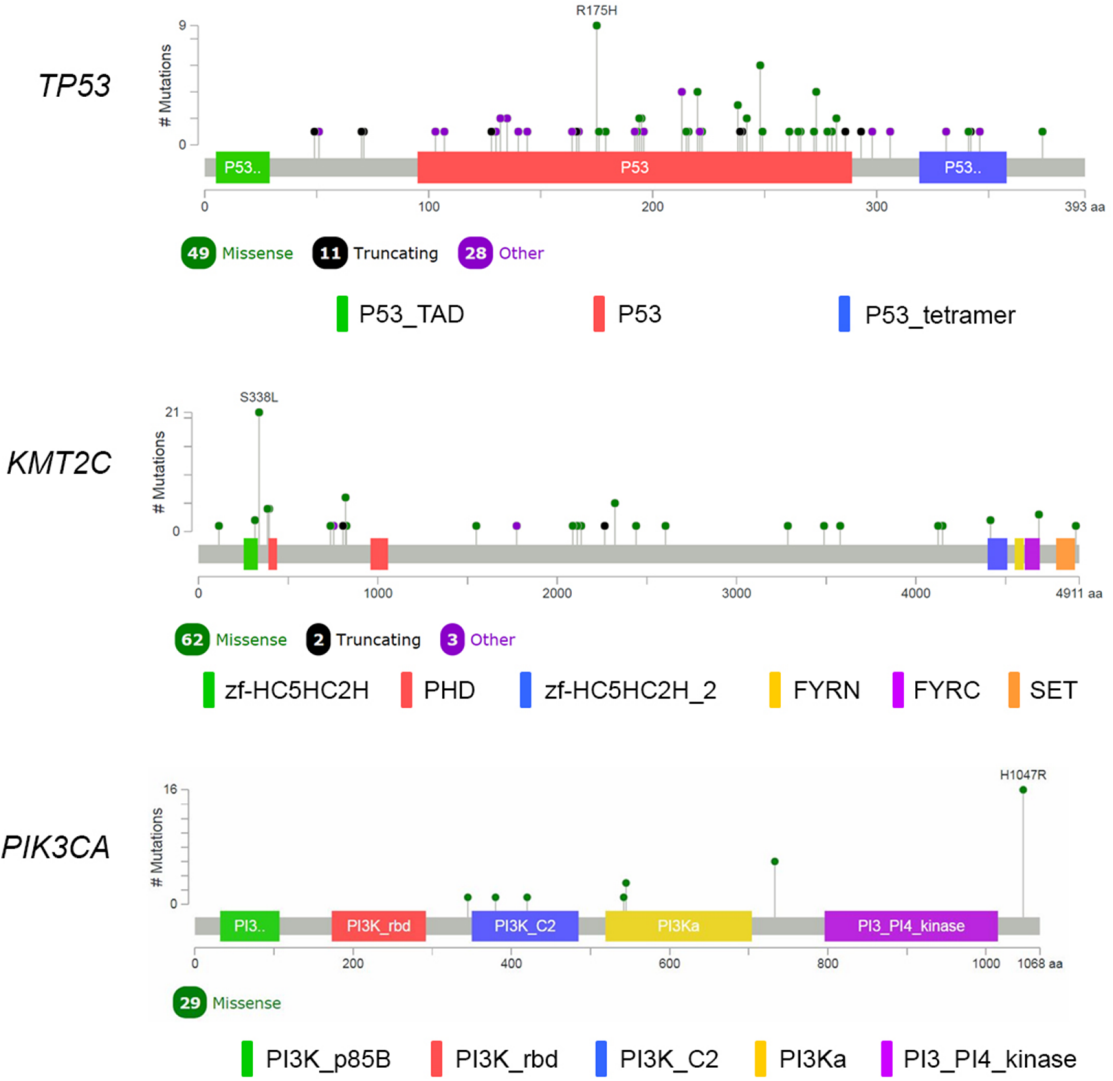
Mutational spectrum of *TP53, KMT2C* and *PIK3CA* in TNBC. The images showed protein domains and the positions of specific mutations with most common type of mutations in each genes labeled. A black dot indicated a truncating mutation; a green dot indicated a missense mutation; and a purple dot indicated other types of mutation.

We performed analysis of the filtered cancer genes to identify potential genes of interest. We first examined for genes mutated in multiple samples and found that 14 genes were mutated in at least 10% of the samples. *SYNE1* was excluded from analysis due to unclear role in cancer. *TP53* was the most frequently mutated gene with variants found in 88 samples. *KMT2C*, which encodes for the lysine methyltransferase mixed-lineage leukemia (MLL3), was the next most frequently mutated gene with mutations in 67 samples. The third most frequently mutated gene was *PIK3CA*, which was shared by 27 samples. Together, 111 of the 116 samples had an alteration in at least *TP53, KMT2C* or *PIK3CA* (Fig. 2). The most commonly recurring mutation was the *PIK3CA* H1074R mutation, which was found in the majority of the *PIK3CA* mutant samples (16 out of 27 samples; 59%). Within these genes, we also found a number of recurrent variants including the previously reported mutations in *KMT2C* S338L (21 samples; 31%), *TP53* R175H (10 samples; 11%), R248Q (five samples; 6%), Y220C (four samples; 5%) and R273H (four samples; 5%) and *PIK3CA* K733R (six samples; 22%) and E545K (four samples; 15%) (Fig. 3).

### *TP53* mutation status and co-occurring mutations

As previously shown, *TP53* was the most commonly mutated gene in the cohort with 88 of the 116 samples (76%) containing a mutation. To identify roles of *TP53* and its association with the other two most commonly mutated genes; *KMT2C* and *PIK3CA*, we subdivided the cohort as *TP53* wild-type and *TP53* mutant groups. In the *TP53* wild-type samples (*n* = 28), the PI3K pathway appeared to be a predominant driver with 19 samples (68%) containing a mutation in either the *PIK3CA* gene or PI3K pathway members, including *PIK3C2B, PIK3CG* and *PTEN*. In the *TP53* mutant group, 67 samples (76%) had one or more mutations in genes encoding chromatin remodeling proteins, including *ATRX, DNMT3A* and *KMT2C*, which have been reported to be involved in cancer. This mutation co-occurrence may suggest a complex interplay in TNBC. However, there was no association between *TP53, PIK3CA* or *KMT2C* mutation status and cancer staging.

### Tumor mutation burden

One of the biological hallmarks in cancer is genome instability. Genome alterations, which either involve in carcinogenesis or occur as a result of widespread genome instability, can create neoantigens and trigger immune response. We used the variant data to calculate the tumor mutation burden for each sample and employed the same filtering scheme accounted for all variants including both nonsynonymous and synonymous calls. Tumor mutation burden was defined as the number of somatic base substitutions, and indels per megabase of coding genome sequence examined. Synonymous mutations are counted in order to reduce sampling noise. Though majority of synonymous mutations are not likely to cause tumor immunogenicity, they may reflect mutational processes that will also result in nonsynonymous mutations and neoantigens elsewhere in the genome (Diederichs et al., 2016; Supek et al., 2014). We found that Thai TNBC has an average tumor mutation 7.3 variants per megabase (95% CI [6.9–7.6]), which consists of nonsynonymous mutation 3.9 variants per megabase (95% CI [3.7–4.1]) and synonymous mutation 3.4 variants per megabase (95% CI [3.3–3.6]). This data showed significantly higher tumor mutation burden than median value (3.6 variants per megabase) found in invasive ductal breast carcinoma cohort (Chalmers et al., 2017).

## Discussion

Triple negative breast cancer is a heterogeneous disease with marked variation in clinical characteristics and response to treatment (Blows et al., 2010). Genome data from previous studies confirmed spectrum of mutational profiles in TNBC are diverse between each patients and cohorts (Cancer Genome Atlas Network, 2012; Pereira et al., 2016; Shah et al., 2012; Stephens et al., 2012). Though there are several explanations for diverse genome landscape in TNBC, patient’s ethnicity could play a significant role in this discrepancy. For the first time, this study provided the mutation profile of TNBC from Thai breast cancer patients. The data from our cohort showed that, besides *TP53* which is the most frequently mutated gene in TNBC, Thai TNBC patients have much higher mutation frequencies in many cancer genes than Western patients. This increase could be due to its exceptionally different pattern of somatic genome alterations in Thai TNBC or its representation of higher tumor mutation burden that occurs extensively throughout cancer genome or both.

*TP53* remains the most commonly mutated gene in Thai TNBC similar to other reported studies (Lefebvre et al., 2016; Cancer Genome Atlas Network, 2012; Pereira et al., 2016). This gene is widely considered guardian of the genome due to its crucial function in maintaining genome integrity, regulating cell cycle and initiating apoptosis. Numerous types of mutations in *TP53* are found in various cancers and mutations occur throughout the entire *TP53* as expected in loss-of-function (LOF) mutations in tumor suppressor gene. We also identify several recurring mutations in *TP53* including R175H, Y220C, R248Q and R273H, which together account for 26% of *TP53* mutant group. These four mutations are hotspot mutations that believed to be oncogenic missense variants (Soussi & Wiman, 2015). Previous study in breast cancer showed that oncogenic *TP53* variants in DNA binding domain (amino acid position 102-292) are associated with reduced survival compared to wild-type *TP53* with an exception of Y220C, which is associated with better survival in breast cancer (Olivier et al., 2006). However, such association could not be identified likely due to very few mortality during our 3-year follow-up period.

*PIK3CA* mutations are usually enriched in hormonal receptor-positive tumor at 29–45%, with lower frequency in TNBC (Cancer Genome Atlas Network, 2012). *PIK3CA* is the most commonly mutated oncogene in Thai TNBC cohort similar to other breast cancer studies. Nevertheless, the mutation frequency of *PIK3CA* in Thai TNBC is much higher than previously published data (Cancer Genome Atlas Network, 2012; Pereira et al., 2016; Shah et al., 2012). H1074R is the major *PIK3CA* hotspot mutation followed by K733R and E545K. These variants are known oncogenic gain-of-function mutations found in multiple types of cancers. Together with other PI3K pathway members, TNBC harboring mutations in *PIK3CA* and its related genes could be potential targets for PI3K inhibitors.

*BRCA1* and *BRCA2* encode proteins that become parts of a complex that repairs double-strand DNA breaks. They are critical for maintaining genome integrity. Breast cancers occurred in most germline *BRCA1* mutation carriers are TNBC. By contrast, there is no characteristic breast cancer subtype in *BRCA2* carriers (Atchley et al., 2008). Most TNBC patients do not harbor germline *BRCA1* or *BRCA2* mutations. However, pathological high-grade breast cancers and TNBC often showed somatic mutations or abnormal expression of *BRCA1* or *BRCA2* (von Wahlde et al., 2017). Thai TNBC displayed high prevalence of somatic alterations in both genes. By comparison with three Western cohorts, the difference was less obvious because the mutation frequencies did vary from one cohort to another. Nevertheless, defects in genome repair machinery related to *BRCA1* and *BRCA2* mutations could make treatment with platinum-based chemotherapy or PARP inhibitors more effective in those patients (Papadimitriou, Mountzios & Papadimitriou, 2018; Vollebergh et al., 2014).

The *KMT2C* is noticeable because it is the second most commonly mutated gene (58%) in our Thai TNBC cohort. *KMT2C* is a gene in myeloid/lymphoid or MLL family and encodes MLL3 lysine-specific histone methyltransferase enzyme. H3 'Lys-4' methylation in histone by the enzyme represents a specific tag for epigenetic transcriptional activation (Rao & Dou, 2015). LOF mutations in *KMT2C* are found in myeloid leukemia, melanoma, glioblastoma multiforme, hepatocellular carcinoma, esophageal cancer, colorectal cancer and pancreatic cancer (Fujimoto et al., 2012; García-Sanz et al. 2017; Liu et al., 2015; Lu et al., 2016; Song et al., 2014). Though the biological role of MLL3 histone methyltransferase in carcinogenesis remains unknown, LOF mutations and downregulation of this gene in cancers suggest that *KMT2C* may act as tumor suppressor gene (Xia et al., 2015). *KMT2C* mutations are also reported in TNBC (Liu et al., 2015). However, Thai TNBC demonstrates substantially higher prevalence of *KMT2C* mutations than TNBC data from Caucasian population (Cancer Genome Atlas Network, 2012; Pereira et al., 2016). A study from African American TNBC also displayed higher *KMT2C* mutations than Caucasian patients (Ademuyiwa et al., 2017). A recently published data identified *KMT2C* mutations in 21% of breast cancer patients from Singapore and Korea (Yap et al., 2018). Interestingly, *KMT2C* has one identified hotspot variant; S338L (31%) which has been previously reported in colorectal cancer (Lu et al., 2016). This observation suggests that epigenetic change may contribute to the development of TNBC and play significant role in Thai TNBC patients. This data could lead to a new insight on epigenetic role of breast carcinogenesis. Further investigation is warranted to provide better understanding on mechanisms of *KMT2C* and a novel treatment strategy.

As previously mentioned, Thai TNBC has generally higher mutation burden than breast cancer in Western patients. The reason for this finding remains not fully explained. Previous study showed that a subset of TNBC harbors somatic mutations in genome repair system (Shah et al., 2012; Stephens et al., 2012). Higher tumor mutational load was observed in hormonal receptor negative than hormonal receptor-positive breast cancer (Barrett et al., 2018; Haricharan et al., 2014). Early phase of anti-PD-1 clinical trials also showed higher response rate in TNBC than hormonal receptor-positive breast cancer (Nanda et al., 2016; Rugo et al., 2018). Our finding suggests that immunotherapy could provide benefit to some Thai TNBC patients.

In this study, the data from Thai TNBC would contribute much-needed information from Asian patients to breast cancer genome landscape and provide another evidence on role of KMT2C in breast carcinogenesis. However, our study has three major limitations. First, only patients from Siriraj and King Chulalongkorn Memorial Hospitals were recruited. Both Bangkok-based university hospitals were the largest hospitals in Thailand and served as major referral centers of Thailand’s healthcare system. Though breast cancer patients treated at our hospitals came from all over the country, many patients from other regions of Thailand who could be treated locally did not participate in the study. The data may not represent the whole picture of Thai TNBC patients. Second, the follow-up period up to 3 years was not long enough to observe clinically meaningful association between genomic alterations and clinical outcomes. Further study would be required. Third, genome sequencing was done on only tumor gDNA extracted from FFPE samples. It has been recognized that the quality of gDNA from FFPE is lower than fresh samples and potentially causes variant call discrepancy. Our study chose to focus on list of cancer-associated genes and apply variant call only when genomic regions have sufficient sequencing depth. This approach has shown to minimize erroneous variant calls, improve precision and acceptable correlation with matched normal-tumor pair sequencing (Chalmers et al., 2017; Oh et al., 2015; Teer et al., 2017). Nevertheless, comparison of our TMB data with previously published studies could be limited by differences in study designs and data analysis methods.

## Conclusion

In conclusion, this study is the first cohort of Thai TNBC patients that demonstrated a distinctive genome alterations including higher mutational burden, higher mutation frequencies on several cancer-associated genes and mutations in *KMT2C*. These results support the genomic heterogeneity between Caucasian and Thai TNBC and could present the new therapeutic approach on histone modification and immunotherapy in TNBC patients. Further investigation is warranted to provide better understanding on role of KMT2C in breast carcinogenesis.

## Supplemental Information

10.7717/peerj.6501/supp-1 Supplemental Information 1 Mutational data on most commonly mutated genes found in each sample of Thai TNBC cohort. Each tab represented mutational data on most commonly mutated genes found in each sample of Thai TNBC cohort, designated by gene name.

## Abbreviations

BMI: Body mass index
CADD: Combined annotation dependent depletion
COSMIC: Catalogue of somatic mutations in cancer
DNA: Deoxyribonucleic acid
ER: Estrogen receptor
ExAC: Exome aggregation database
FFPE: Formalin-fixed, paraffin-embedded
gDNA: Genomic deoxyribonucleic acid
HER2: Human epidermal growth factor receptor 2
HGMD: Human gene mutation database
LOF: Loss of function
LRT: Likelihood ratio test
OMIM: Online Mendelian inheritance in men
PAM50: Prosigna intrinsic subtype of breast cancer
PARP: Poly (ADP-ribose) polymerase
PI3K: Phosphatidylinositide 3-kinases
PR: Progesterone receptor
SIFT: Sorting intolerance from tolerance
SNP: Single nucleotide polymorphism
SO: Sequence ontology
TCGA: The cancer genome atlas
TNBC: Triple negative breast cancer
UTR: Untranslated region

## Additional Information and Declarations

### Data Availability

The following information was supplied regarding the deposition of DNA sequences: The sequence data of this study are accessible via Figshare: Suktitipat, Bhoom; Pithukpakorn, Manop (2018): Triple Negative Breast Cancer Data. figshare. Fileset. DOI 10.6084/m9.figshare.7467401.v2 NCBI BioProject database, ID PRJNA514873.

## References

[ref-1] Ademuyiwa FO, Tao Y, Luo J, Weilbaecher K, Ma CX (2017). Differences in the mutational landscape of triple-negative breast cancer in African Americans and Caucasians. Breast Cancer Research and Treatment.

[ref-2] Atchley DP, Albarracin CT, Lopez A, Valero V, Amos CI, Gonzalez-Angulo AM, Hortobagyi GN, Arun BK (2008). Clinical and pathologic characteristics of patients with *BRCA*-positive and *BRCA*-negative breast cancer. Journal of Clinical Oncology.

[ref-3] Banegas MP, Li CI (2012). Breast cancer characteristics and outcomes among Hispanic Black and Hispanic White women. Breast Cancer Research and Treatment.

[ref-4] Barrett MT, Lenkiewicz E, Malasi S, Basu A, Yearley JH, Annamalai L, McCullough AE, Kosiorek HE, Narang P, Wilson Sayres MA, Chen M, Anderson KS, Pockaj BA (2018). The association of genomic lesions and PD-1/PD-L1 expression in resected triple-negative breast cancers. Breast Cancer Research.

[ref-5] Blows FM, Driver KE, Schmidt MK, Broeks A, van Leeuwen FE, Wesseling J, Cheang MC, Gelmon K, Nielsen TO, Blomqvist C, Heikkilä P, Heikkinen T, Nevanlinna H, Akslen LA, Bégin LR, Foulkes WD, Couch FJ, Wang X, Cafourek V, Olson JE, Baglietto L, Giles GG, Severi G, McLean CA, Southey MC, Rakha E, Green AR, Ellis IO, Sherman ME, Lissowska J, Anderson WF, Cox A, Cross SS, Reed MW, Provenzano E, Dawson SJ, Dunning AM, Humphreys M, Easton DF, García-Closas M, Caldas C, Pharoah PD, Huntsman D (2010). Subtyping of breast cancer by immunohistochemistry to investigate a relationship between subtype and short and long term survival: a collaborative analysis of data for 10,159 cases from 12 studies. PLOS Medicine.

[ref-20] Cancer Genome Atlas Network (2012). Comprehensive molecular portraits of human breast tumours. Nature.

[ref-6] Chalmers ZR, Connelly CF, Fabrizio D, Gay L, Ali SM, Ennis R, Schrock A, Campbell B, Shlien A, Chmielecki J, Huang F, He Y, Sun J, Tabori U, Kennedy M, Lieber DS, Roels S, White J, Otto GA, Ross JS, Garraway L, Miller VA, Stephens PJ, Frampton GM (2017). Analysis of 100,000 human cancer genomes reveals the landscape of tumor mutational burden. Genome Medicine.

[ref-7] Chen L, Li CI (2015). Racial disparities in breast cancer diagnosis and treatment by hormone receptor and HER2 status. Cancer Epidemiology Biomarkers & Prevention.

[ref-8] Diederichs S, Bartsch L, Berkmann JC, Fröse K, Heitmann J, Hoppe C, Iggena D, Jazmati D, Karschnia P, Linsenmeier M, Maulhardt T, Möhrmann L, Morstein J, Paffenholz SV, Röpenack P, Rückert T, Sandig L, Schell M, Steinmann A, Voss G, Wasmuth J, Weinberger ME, Wullenkord R (2016). The dark matter of the cancer genome: aberrations in regulatory elements, untranslated regions, splice sites, non‐coding RNA and synonymous mutations. EMBO Molecular Medicine.

[ref-9] Fujimoto A, Totoki Y, Abe T, Boroevich KA, Hosoda F, Nguyen HH, Aoki M, Hosono N, Kubo M, Miya F, Arai Y, Takahashi H, Shirakihara T, Nagasaki M, Shibuya T, Nakano K, Watanabe-Makino K, Tanaka H, Nakamura H, Kusuda J, Ojima H, Shimada K, Okusaka T, Ueno M, Shigekawa Y, Kawakami Y, Arihiro K, Ohdan H, Gotoh K, Ishikawa O, Ariizumi S-I, Yamamoto M, Yamada T, Chayama K, Kosuge T, Yamaue H, Kamatani N, Miyano S, Nakagama H, Nakamura Y, Tsunoda T, Shibata T, Nakagawa H (2012). Whole-genome sequencing of liver cancers identifies etiological influences on mutation patterns and recurrent mutations in chromatin regulators. Nature Genetics.

[ref-10] Gao J, Aksoy BA, Dogrusoz U, Dresdner G, Gross B, Sumer SO, Sun Y, Jacobsen A, Sinha R, Larsson E, Cerami E, Sander C, Schultz N (2013). Integrative analysis of complex cancer genomics and clinical profiles using the cBioPortal. Science Signaling.

[ref-11] García-Sanz P, Triviño JC, Mota A, Pérez López Mía, Colás E, Rojo-Sebastián A, García Ángel, Gatius S, Ruiz Mía, Prat J, López-López R, Abal M, Gil-Moreno A, Reventós J, Matias-Guiu X, Moreno-Bueno G (2017). Chromatin remodelling and DNA repair genes are frequently mutated in endometrioid endometrial carcinoma. International Journal of Cancer.

[ref-12] Haricharan S, Bainbridge MN, Scheet P, Brown PH (2014). Somatic mutation load of estrogen receptor-positive breast tumors predicts overall survival: an analysis of genome sequence data. Breast Cancer Research and Treatment.

[ref-13] Huo D, Hu H, Rhie SK, Gamazon ER, Cherniack AD, Liu J, Yoshimatsu TF, Pitt JJ, Hoadley KA, Troester M, Ru Y, Lichtenberg T, Sturtz LA, Shelley CS, Benz CC, Mills GB, Laird PW, Shriver CD, Perou CM, Olopade OI (2017). Comparison of breast cancer molecular features and survival by African and European ancestry in the cancer genome atlas. JAMA Oncology.

[ref-14] Lefebvre C, Bachelot T, Filleron T, Pedrero M, Campone M, Soria J-C, Massard C, Lévy C, Arnedos M, Lacroix-Triki M, Garrabey J, Boursin Y, Deloger M, Fu Y, Commo F, Scott V, Lacroix L, Dieci MV, Kamal M, Diéras V, Gonçalves A, Ferrerro J-M, Romieu G, Vanlemmens L, Mouret Reynier M-A, Théry J-C, Le Du F, Guiu S, Dalenc F, Clapisson G, Bonnefoi H, Jimenez M, Le Tourneau C, André F, Mardis ER (2016). Mutational profile of metastatic breast cancers: a retrospective analysis. PLOS Medicine.

[ref-15] Liu L, Kimball S, Liu H, Holowatyj A, Yang Z-Q (2015). Genetic alterations of histone lysine methyltransferases and their significance in breast cancer. Oncotarget.

[ref-16] Lu Y-W, Zhang H-F, Liang R, Xie Z-R, Luo H-Y, Zeng Y-J, Xu Y, Wang L-M, Kong X-Y, Wang K-H, Suzuki H (2016). Colorectal cancer genetic heterogeneity delineated by multi-region sequencing. PLOS ONE.

[ref-17] Malorni L, Shetty PB, De Angelis C, Hilsenbeck S, Rimawi MF, Elledge R, Osborne CK, De Placido S, Arpino G (2012). Clinical and biologic features of triple-negative breast cancers in a large cohort of patients with long-term follow-up. Breast Cancer Research and Treatment.

[ref-18] McLaren W, Gil L, Hunt SE, Riat HS, Ritchie GRS, Thormann A, Flicek P, Cunningham F (2016). The ensembl variant effect predictor. Genome Biology.

[ref-19] Nanda R, Chow LQM, Dees EC, Berger R, Gupta S, Geva R, Pusztai L, Pathiraja K, Aktan G, Cheng JD, Karantza V, Buisseret L (2016). Pembrolizumab in patients with advanced triple-negative breast cancer: phase Ib KEYNOTE-012 study. Journal of Clinical Oncology.

[ref-21] Oh E, Choi Y-L, Kwon MJ, Kim RN, Kim YJ, Song J-Y, Jung KS, Shin YK, Castresana JS (2015). Comparison of accuracy of whole-exome sequencing with formalin-fixed paraffin-embedded and fresh frozen tissue samples. PLOS ONE.

[ref-22] Olivier M, Langerod A, Carrieri P, Bergh J, Klaar S, Eyfjord J, Theillet C, Rodriguez C, Lidereau R, Bieche I, Varley J, Bignon Y, Uhrhammer N, Winqvist R, Jukkola-Vuorinen A, Niederacher D, Kato S, Ishioka C, Hainaut P, Borresen-Dale AL (2006). The clinical value of somatic TP53 gene mutations in 1,794 patients with breast cancer. Clinical Cancer Research.

[ref-23] Papadimitriou M, Mountzios G, Papadimitriou CA (2018). The role of PARP inhibition in triple-negative breast cancer: unraveling the wide spectrum of synthetic lethality. Cancer Treatment Reviews.

[ref-24] Pereira B, Chin S-F, Rueda OM, Vollan H-KM, Provenzano E, Bardwell HA, Pugh M, Jones L, Russell R, Sammut S-J, Tsui DWY, Liu B, Dawson S-J, Abraham J, Northen H, Peden JF, Mukherjee A, Turashvili G, Green AR, McKinney S, Oloumi A, Shah S, Rosenfeld N, Murphy L, Bentley DR, Ellis IO, Purushotham A, Pinder SE, Børresen-Dale A-L, Earl HM, Pharoah PD, Ross MT, Aparicio S, Caldas C (2016). The somatic mutation profiles of 2,433 breast cancers refines their genomic and transcriptomic landscapes. Nature Communications.

[ref-25] Rao RC, Dou Y (2015). Hijacked in cancer: the KMT2 (MLL) family of methyltransferases. Nature Reviews Cancer.

[ref-26] Rhee J, Han S-W, Oh D-Y, Kim JH, Im S-A, Han W, Ae Park I, Noh D-Y, Bang Y-J, Kim T-Y (2008). The clinicopathologic characteristics and prognostic significance of triple-negativity in node-negative breast cancer. BMC Cancer.

[ref-27] Rugo HS, Delord J-P, Im S-A, Ott PA, Piha-Paul SA, Bedard PL, Sachdev J, Tourneau CL, van Brummelen EMJ, Varga A, Salgado R, Loi S, Saraf S, Pietrangelo D, Karantza V, Tan AR (2018). Safety and antitumor activity of pembrolizumab in patients with estrogen receptor–positive/human epidermal growth factor receptor 2–negative advanced breast cancer. Clinical Cancer Research.

[ref-28] Shah SP, Roth A, Goya R, Oloumi A, Ha G, Zhao Y, Turashvili G, Ding J, Tse K, Haffari G, Bashashati A, Prentice LM, Khattra J, Burleigh A, Yap D, Bernard V, McPherson A, Shumansky K, Crisan A, Giuliany R, Heravi-Moussavi A, Rosner J, Lai D, Birol I, Varhol R, Tam A, Dhalla N, Zeng T, Ma K, Chan SK, Griffith M, Moradian A, Cheng SW, Morin GB, Watson P, Gelmon K, Chia S, Chin SF, Curtis C, Rueda OM, Pharoah PD, Damaraju S, Mackey J, Hoon K, Harkins T, Tadigotla V, Sigaroudinia M, Gascard P, Tlsty T, Costello JF, Meyer IM, Eaves CJ, Wasserman WW, Jones S, Huntsman D, Hirst M, Caldas C, Marra MA, Aparicio S (2012). The clonal and mutational evolution spectrum of primary triple-negative breast cancers. Nature.

[ref-29] Song Y, Li L, Ou Y, Gao Z, Li E, Li X, Zhang W, Wang J, Xu L, Zhou Y, Ma X, Liu L, Zhao Z, Huang X, Fan J, Dong L, Chen G, Ma L, Yang J, Chen L, He M, Li M, Zhuang X, Huang K, Qiu K, Yin G, Guo G, Feng Q, Chen P, Wu Z, Wu J, Ma L, Zhao J, Luo L, Fu M, Xu B, Chen B, Li Y, Tong T, Wang M, Liu Z, Lin D, Zhang X, Yang H, Wang J, Zhan Q (2014). Identification of genomic alterations in oesophageal squamous cell cancer. Nature.

[ref-30] Soussi T, Wiman KG (2015). TP53: an oncogene in disguise. Cell Death & Differentiation.

[ref-31] Stephens PJ, Tarpey PS, Davies H, Van Loo P, Greenman C, Wedge DC, Nik-Zainal S, Martin S, Varela I, Bignell GR, Yates LR, Papaemmanuil E, Beare D, Butler A, Cheverton A, Gamble J, Hinton J, Jia M, Jayakumar A, Jones D, Latimer C, Lau KW, McLaren S, McBride DJ, Menzies A, Mudie L, Raine K, Rad R, Chapman MS, Teague J, Easton D, Langerod A, Lee MT, Shen CY, Tee BT, Huimin BW, Broeks A, Vargas AC, Turashvili G, Martens J, Fatima A, Miron P, Chin SF, Thomas G, Boyault S, Mariani O, Lakhani SR, van de Veer M, van 't Vijver L, Foekens J, Desmedt C, Sotiriou C, Tutt A, Caldas C, Reis-Filho JS, Aparicio SA, Salomon AV, Borresen-Dale AL, Richardson AL, Campbell PJ, Futreal PA, Stratton MR, The Oslo Breast Cancer Consortium (OSBREAC) (2012). The landscape of cancer genes and mutational processes in breast cancer. Nature.

[ref-32] Supek F, Minana B, Valcárcel J, Gabaldón T, Lehner B (2014). Synonymous mutations frequently act as driver mutations in human cancers. Cell.

[ref-33] Teer JK, Zhang Y, Chen L, Welsh EA, Cress WD, Eschrich SA, Berglund AE (2017). Evaluating somatic tumor mutation detection without matched normal samples. Human Genomics.

[ref-34] Thike AA, Cheok PY, Jara-Lazaro AR, Tan B, Tan P, Tan PH (2009). Triple-negative breast cancer: clinicopathological characteristics and relationship with basal-like breast cancer. Modern Pathology.

[ref-35] Vollebergh MA, Lips EH, Nederlof PM, Wessels LFA, Wesseling J, vd Vijver MJ, de Vries EGE, van Tinteren H, Jonkers J, Hauptmann M, Rodenhuis S, Linn SC (2014). Genomic patterns resembling BRCA1- and BRCA2-mutated breast cancers predict benefit of intensified carboplatin-based chemotherapy. Breast Cancer Research.

[ref-36] von Wahlde M-K, Timms KM, Chagpar A, Wali VB, Jiang T, Bossuyt V, Saglam O, Reid J, Gutin A, Neff C, Lanchbury JS, Hatzis C, Hofstatter E, Pusztai L (2017). Intratumor heterogeneity of homologous recombination deficiency in primary breast cancer. Clinical Cancer Research.

[ref-37] Xia M, Xu L, Leng Y, Gao F, Xia H, Zhang D, Ding X (2015). Downregulation of MLL3 in esophageal squamous cell carcinoma is required for the growth and metastasis of cancer cells. Tumor Biology.

[ref-38] Yap Y-S, Singh AP, Lim JHC, Ahn J-H, Jung K-H, Kim J, Dent RA, Ng RCH, Kim S-B, Chiang DY (2018). Elucidating therapeutic molecular targets in premenopausal Asian women with recurrent breast cancers. npj Breast Cancer.

